# Living Life Through Sport: The Transition of Elite Spanish Student-Athletes to a University Degree in Physical Activity and Sports Sciences

**DOI:** 10.3389/fpsyg.2020.01367

**Published:** 2020-06-23

**Authors:** Pau Mateu, Eduard Inglés, Miquel Torregrossa, Renato Francisco Rodrigues Marques, Natalia Stambulova, Anna Vilanova

**Affiliations:** ^1^Institut Nacional d’Educació Física de Catalunya (INEFC), Universitat de Barcelona, Barcelona, Spain; ^2^Departament de Psicologia Bàsica, Universitat Autònoma de Barcelona, Barcelona, Spain; ^3^Institut de Recerca de l’Esport, Universitat Autònoma de Barcelona, Barcelona, Spain; ^4^School of Physical Education and Sport of Ribeirão Preto, University of São Paulo, Ribeirão Preto, Brazil; ^5^Akademin för hälsa och välfärd, Högskolan i Halmstad, Halmstad, Sweden

**Keywords:** transition, dual career, elite sport, higher education, sport sciences

## Abstract

Interest in studying the different transitions faced by elite athletes throughout their careers has grown significantly in recent years. While transition from secondary school to university is an important research area in Europe, there is a void of studies on how student-athletes experience the transition to specific degrees. One of the most sought-after university degrees among elite athletes in Spain is a degree in Physical Activity and Sport Sciences (PASS). The first aim of this study was to investigate the main demands, barriers, and resources perceived by elite student-athletes in various phases of dual career transition to a university degree in PASS. The second aim was to identify the transition pathways pursued depending on the subjective importance they attached to sport and education. Eleven elite student-athletes (*M*_age_ = 20.7, SD = 1.6 years) who were in their second and third year of the degree in PASS participated in semi-structured interviews. Deductive-inductive thematic analysis of the interview transcripts revealed three main themes: (a) general university transition issues, (b) PASS-specific transition issues, and (c) transition pathways. Our results show that the close link between sport and the content of the degree was perceived by the elite student-athletes as their main resource. This link, however, was also perceived as a major barrier as the compulsory practical subjects entailed a risk of injury or overtraining that could affect both athletic and academic development. We noticed how the importance they attached to sport or studies varied at different moments of the transition period, a phenomenon we termed “fluid transition pathways.” Dual career promotion for elite athletes is an important part of European sports policy, and our findings provide new knowledge that could help Spanish PASS faculties develop specific assistance programs to support transitioning student-athletes.

## Introduction

In recent years, there has been growing interest in the career transitions experienced by elite athletes at different moments and in different spheres of their lives, such as the transition from junior to senior competition (Chamorro et al., 2016; Torregrossa et al., 2016; Stambulova et al., 2017) and the transition from sport to an alternative career in the wider labor market (Torregrossa et al., 2015; Vilanova and Puig, 2017; Brown et al., 2018).

Career transitions have been defined as “turning phases or shifts in athletes’ development associated with a set of specific demands that athletes have to cope with in order to continue successfully in sport and/or other spheres of their life” (Stambulova and Wylleman, 2014, p. 607). According to the holistic athletic career model (Wylleman et al., 2013), elite athletes develop their careers and experience transitions in a range of sporting and non-sporting contexts encompassing athletic, psychological, psychosocial, academic, and financial domains. Transitions can be normative, non-normative, or quasi-normative. Normative transitions are relatively predictable (e.g., transition from junior to senior sport), while non-normative transitions are unpredictable (e.g., transition due to injury) (Stambulova et al., 2009) and quasi-normative transitions are predictable for certain groups of athletes (e.g., transition to non-compulsory education, cultural transitions) (Stambulova, 2016). The ability to predict normative and quasi-normative transitions creates an opportunity to prepare athletes to face them in advance (Alfermann and Stambulova, 2007).

The concept of a dual career, combining sport and education or work, is a growing research area (Aquilina, 2013; Stambulova and Wylleman, 2019). Transition from secondary education to university is a quasi-normative, non-sports-related transition that has been shown to be of great importance in the life of elite student-athletes (ESAs) (Tekavc et al., 2015; Miró et al., 2018). It forms part of the academic-vocational development of ESAs who decide to pursue a dual career in sport and education after secondary school (Aquilina and Henry, 2010; Wylleman et al., 2013; De Brandt, 2017). In a longitudinal study of Spanish ESAs, Pérez-Rivasés et al. (2017) identified four phases within this transition: (a) preparation (last year of secondary school up to university entrance exams), (b) immersion (first contact with university up to first exams), (c) learning (beginning of second semester up to end of first academic year), and (d) adaptation (entire second year).

Transition to higher education among elite athletes has been addressed in numerous studies, many of which have been conducted in the United States (Tracey and Corlett, 1995; Petrie and Stoever, 1997; Giacobbi et al., 2004; Petitpas et al., 2009; Naphy, 2016). In Europe, studies on transitions between different levels of education have provided useful insights into problems associated with these life changes (Stambulova and Wylleman, 2014). Researchers from numerous countries, including the United Kingdom (McKenna and Dunstan-Lewis, 2004; Brown et al., 2015), Belgium (De Brandt, 2017; De Brandt et al., 2018), Slovakia (Geraniosova and Ronkainen, 2015), Italy (Guidotti et al., 2015; Lupo et al., 2017a, b; Brustio et al., 2019), and Spain (Álvarez and López, 2012; Pérez-Rivasés et al., 2017; Mateu et al., 2018b; Miró et al., 2018), have analyzed multiple aspects of transitioning from school to university among ESAs, such as the demands of combining sport and study, the importance of effective dual career assistance systems and competencies such as time management and planning, gender differences in academic qualifications, and perceptions of transitional experiences.

A number of explanatory models have been developed over the years to help define the processes, outcomes, and factors associated with transitions (Schlossberg, 1981; Taylor and Ogilvie, 1994; Stambulova, 2003). The athletic career transition model proposed by Stambulova (2003) is particularly interesting, as it helps explain the different types of transition that may occur in an athlete’s career, including transitions into new educational settings (Brown et al., 2015; Stambulova et al., 2015). Stambulova’s (2003) model considers transitions to be processes in which athletes must apply different strategies to cope with a series of demands. The effectiveness of these strategies will depend on existing resources and barriers (internal and external factors that facilitate or hinder the coping process). The transition is considered to be successful when the athletes overcome the demands they face; if they are unable to cope, they will experience what Stambulova calls a “crisis transition” and may require psychological support.

Athletic identity is another important factor in the study of career transitions of athletes. This concept has been defined as the degree to which an individual identifies with the athlete role and the importance they give to this life sphere over others (Brewer et al., 1993; Lally, 2007). National and international studies and reviews of career termination in sport have established that athletic identity influences how an athlete adapts to and perceives transition. A high level of athletic identity has been associated with adaptation difficulties and negative emotions and perceptions (Brewer et al., 2000; Alfermann and Stambulova, 2007; Lavallee and Robinson, 2007; Pallarés et al., 2011). Pallarés et al. (2011) described three elite athlete career pathways that varied according to the priority placed on sport and education: (a) a linear path, where sport only is prioritized; (b) a convergent path, where sport is prioritized but combined with education; and (c) a parallel path, where sport and education are given equal priority. According to Miró et al. (2018), ESAs need to have a multidimensional (i.e., athletic, academic, and other) identity if they are to better maintain a dual career in sport and education after secondary school, and having this multidimensional identity offers greater guarantees of transition success. Studies conducted in the United States (Cox et al., 2009) and Taiwan (Huang et al., 2016) have not found athletic identity to be a determining factor in how ESAs perceive barriers in the university environment.

It is not uncommon for elite athletes who decide to go to university to choose to study Physical Activity and Sport Sciences (PASS), as this is a field in which they feel particularly comfortable (Honta, 2007). According to Honta, however, this decision may not always be the most appropriate, as the physical requirements of the curriculum may increase fatigue levels. Furthermore, the wide variety of physical and sporting activities that students studying PASS need to perform in order to pass different subjects (Mateu et al., 2018a) might even have a negative effect on athletic performance as elite athletes are typically highly specialized in a given sport (Honta, 2007). Nonetheless, nearly 20% of Spanish Olympic athletes choose to study a PASS degree (cfr. Iríbar, 2003), and in Spain, there is a royal decree that stipulates that universities must reserve 3% of all university places and 5% of all PASS places for elite athletes (Royal Decree 971/2007, 2007).

While each athlete’s career path is unique, common patterns emerge among certain groups of athletes (Alfermann and Stambulova, 2007). A number of studies have examined the transitional experiences of ESAs from specific sports (Gledhill and Harwood, 2015; Tekavc et al., 2015; Guirola et al., 2016; Pink et al., 2018; Gavala-González et al., 2019), but to our knowledge, no studies to date have analyzed the experiences of ESAs enrolled in a specific degree. The first aim of this study was to investigate how ESAs pursuing a dual career in sport and education perceive demands, barriers, and resources during various phases of their transition from school to university to study a degree in PASS. The second aim was to identify the transition pathways followed by the ESAs depending on the subjective importance they attached to sport and education.

## Materials and Methods

### Philosophical Underpinning

This descriptive, retrospective study is framed within the post-positivist paradigm. According to Sparkes and Smith (2014), this position subscribes to realist ontology, that is, it assumes the existence of a single, objective, and understandable external reality, allowing us thus “to formulate rules beyond time and space in order to control and predict” as best as possible (Sparkes and Smith, 2014, p. 11). Lincoln et al. (2011) claim that the ontological position that characterizes post-positivism is critical realism, whereby there is an objective external reality that can only be imperfectly apprehended as observations are fallible, meaning that all theories can be revised. The inherent epistemological approach to post-positivism is a modified dualism/objectivism (Lincoln et al., 2011; Ronkainen et al., 2015) in which researchers are not fully separated from their research. They can get close to the external reality, but they must make efforts to reduce contamination. In other words, they consider that their observations are fallible and that their personal values and positions may shape their understanding of what is being studied.

### Setting

The PASS degree is one of the most highly sought-after degrees in Spain and is offered by over 50 public and private universities. This study was carried out at a PASS faculty with over 730 students at a public university in Barcelona. Barcelona is the city in Spain with a strongest sporting tradition; it hosted the 1992 Summer Olympic Games, and it has important structures supporting the practice of elite sport and the development of elite athletes. The PASS faculty has a dual career assistance program that provides support to approximately 50 ESAs. The program offers several services designed to help elite athletes combine their athletic career and studies. ESAs enrolled in the program are given, for example (a) extended periods for requesting single-test evaluations and can (b) be assessed by continuous evaluation despite not having attended 80% of classes, (c) reschedule evaluation activities (exams, assignments, etc.) due to sporting commitments or injuries, (d) enroll part-time, and (e) switch classes to reconcile academic and training schedules. Each ESA also has a tutor, who is a member of the faculty’s teaching staff. Tutors are responsible for counseling ESAs and mediating with other lecturers in the event of conflict.

### Participants

The participants for this study, who where enrolled in the faculty’s dual career assistance program, were recruited by criterion sampling (Sparkes and Smith, 2014), facilitated by the fact that three of the authors (PM, EI, and AV) are tutors in the program. To be eligible for inclusion, the participants had to (a) be an active elite athlete^1^, (b) be enrolled in the university’s PASS degree, and (c) be in or have recently completed the adaptation phase of transition from secondary to higher education (Pérez-Rivasés et al., 2017) (i.e., they had to be in their second or third year of university). Eleven ESAs (eight men and three women) volunteered to participate in the study and met the selection criteria. The greater presence of men in the sample reflects the distribution of male and female students in the PASS degree in Spain (just 18% of students enrolled in this degree are female) (Serra et al., 2019). At the time of data collection, the ESAs (listed with their pseudonyms in Table 1) were aged between 19 and 24 years (*M*_age_ = 20.7, SD = 1.6). They represented nine different individual or team sports.

**TABLE 1:** Characteristics of the participants.

**Participant**	**Sex**	**Age**	**Year of study**	**Sport**	**Competition level**
Glòria	W	20	3rd	Individual	International
Raquel	W	21	2nd	Team	National
Greta	W	24	3rd	Individual	International
Martí	M	19	2nd	Individual	International
Enric	M	19	2nd	Team	Regional
Pol	M	20	3rd	Individual	International
Joaquim	M	20	2nd	Team	International
Gerard	M	20	2nd	Individual	National
Gustau	M	21	3rd	Team	International
Carles	M	21	2nd	Team	International
Cesc	M	23	3rd	Individual	National

*W, women; M, men.*

### Instruments

We opted to conduct semi-structured interviews for data collection since insights into transition to university will be largely determined by individual experiences and perceptions. Semi-structured interviews give participants greater control than questionnaires or structured interviews (Sparkes and Smith, 2014), as they allow them to tell their story in their own words, without limitations. The main tool used to stimulate contributions was the research diagram, which is a graphic elicitation tool that “may yield contributions from interviewees that are difficult to achieve by verbal exchanges alone” (Crilly et al., 2006). The diagram used in this study was designed based on the theoretical models of Stambulova (2003); Wylleman et al. (2013), and Pérez-Rivasés et al. (2017), and its purpose was to identify the most significant experiences of ESAs studying PASS as they transitioned from secondary school to university. The diagram consisted of an upper timeline indicating the different milestones (e.g., end of last year at secondary school, university entrance exams) along the transition pathway that define the different phases of this process: preparation, immersion, learning, and adaptation (Pérez-Rivasés et al., 2017). The larger central area of the diagram contained two boxes. The box on the left, representing Stambulova’s (2003) concepts of demands, resources, and barriers contained the words “situations experienced,” accompanied by two smaller boxes stating “facilitators” and “difficulties.” To capture information related to the different domains of ESA development (athletic, psychological, psychosocial, academic-vocational, and financial) (Wylleman et al., 2013), the boxes were surrounded by a circular line including the words: “sport,” “feelings,” “people,” “studies/work,” and “money.” On the right, there was a box stating “solution: actions taken, help from others, circumstances” to capture Stambulova’s (2003) concept of coping strategies and other factors that might have helped the ESAs to overcome the demands of transition.

### Procedure

We contacted the participants by email to explain the purpose of the study and the associated ethical and logistical issues. All interviews were conducted in the first semester of the 2018–2019 academic year and were held in different locations to suit the needs and preferences of the participants, although most of them were held on campus. Two actions were taken with the aim of minimizing power asymmetries between the interviewer and interviewees (Anyan, 2013). On the one hand, the interviews were conducted by the first author, who unlike the other authors, who all hold stable faculty positions, is a Ph.D. student closer in age to the participants. On the other hand, the interviews were carried out in a relaxed environment such as the faculty canteen or a quiet bar rather than in more formal office spaces. The interviews lasted an average of 49 min. Before beginning the interviews, the participants were reminded about the aim of the study and about data confidentiality and processing issues. They were given pseudonyms to ensure anonymity. They then read and signed the informed consent documents. All interviews were conducted, tape-recorded, and transcribed verbatim.

### Data Analysis

We conducted a thematic analysis, which is an appropriate method for understanding people’s experiences and perspectives in relation to certain issues (Braun et al., 2016). The data analysis process was both deductive and inductive and consisted of several steps. (1) The authors familiarized themselves with the data by reading and re-reading the transcripts of the interviews. (2) Using the qualitative data analysis software package NVivo 12 (QSR International), we designed a preliminary theory-based proposal for coding the data grounded in the athletic career transition model (Stambulova, 2003), the holistic athletic career model (Wylleman et al., 2013), and the career pathways described by Pallarés et al. (2011) and Miró et al. (2018). As an example, we used the developmental layers described by Wylleman et al. (2013) to distribute the ESAs’ experiences into five domains: athletic, psychological, psychosocial, academic, and financial. (3) We then deductively organized the data into the defined categories. (4) We re-read the structured data and created new codes by means of inductive analysis, taking into account both semantic/explicit and latent/implicit information (Braun et al., 2016). (5) We re-reviewed the data and grouped them into three main themes: general university transition issues, PASS-specific transition issues, and transition pathways. (6) The final step of this process was to produce the research report.

The methodological integrity of our research was guaranteed by following the Reporting Standards for Qualitative Research (American Psychological Association, 2020). Considering our inquiry design and approach, the data collection method (semi-structured interview) and instrument (research diagram) were deemed to be the most suitable tools for capturing relevant information to answer the research questions. In addition, the fact that the study was performed by a multidisciplinary, international team with different levels of academic experience, cultural frameworks, and positions will have helped reduce individual and institutional biases among the three authors who work at the PASS faculty. Discussions among the different members of the team helped with research (re)design and data (re)structuring and (re)interpreting. In addition, meetings and discussions helped us explore our own identities and positioning (e.g., Spanish dual career context connoisseurs vs. connoisseurs of other cultural contexts, PASS faculty workers vs. outsiders). Our findings are evidence-based and we provide relevant quotes from the interviews as proof. The authors’ engagement in the data collection process is described in the data analysis section. As shown in the discussion section, our findings are insightful and meaningful in relation to the literature and our study goal. We also provide contextual information such as the setting of the study and detailed information about participants that is not just limited to descriptions but also provides explanations on issues such as the gender bias present in the sample.

## Results

### General University Transition Issues

Several of the ESAs reported stress and a lack of time to study during the preparation phase of the transition process; they attributed this to their sporting commitments, since they were competing and/or training at more than one level at the same time:

Raquel: In the last year of secondary school, I started playing on the senior team, while still playing junior. I was on both teams. And of course, the last year of secondary school, you know that just after you finish, you have *la selectividad* (Spanish university entrance exams), there is much to study… I had two games every weekend, and it (studying) was complicated because of this. I was in the last year of secondary school, I wanted to study PASS, and the entry grades weren’t exactly low.

These feelings were even more intense in ESAs who also had work commitments:

Carles: Every Saturday morning I played with the junior team, in the first half of the afternoon I competed in swimming, in the second half I played with the senior B water polo team, and in the evening I played with the senior A team. I was busy all Saturday. And on Sundays I had to go to the games of the kids I was training. It was chaos.

The immersion phase was generally perceived as a period with significantly greater academic demands compared with secondary school.

Greta: There was definitely a noticeable change. In fact, I got through secondary school by just going to class and reading my notes the day before each exam. When you start university… you start with anatomy, psychology, sports… it’s a mix, you know? There’s a lot of content, and I got to January (first exams) saying “wow.”

Transition to university usually coincides with other important transitions, such as the transition from junior to senior competition. In our sample, this transition was perceived as more or less challenging depending on the sport, the level at which the ESAs were competing, and the career path they were pursuing:

Cesc: If before you had to make “X” time to qualify for a junior championship, which was already quite demanding, now you have to reduce this time by 5 or 8 s to qualify for a senior championship. It’s an enormous change. It’s not easy. […] When I was at secondary school, qualifying for an international championship was an achievable goal, but now that I’m a senior, I see it as very far away. Whether you like it or not, your motivation drops a little as you suddenly see that you are very far from the minimum qualifying times.
Joaquim: Last year I was still a junior. I moved up to senior this year, but to be honest I haven’t noticed the change because both teams usually train at the same time. I have trained with higher-level teams since I was young, and the training sessions often overlapped. When they didn’t, I stayed on longer and did two sessions in a row, one for my category and one for the next. As a junior I always trained with the senior team, so I haven’t noticed any changes in training load.

An important barrier mentioned by several ESAs in relation to successfully combining their sporting and academic activities was a lack of time to study. As the demands in both areas tended to increase after the transition from school, the ESAs reported that they needed to dedicate more time to achieving their goals in both areas. Not reaching these goals caused them stress and frustration. This perception was shared by athletes involved in team sports and athletes involved in endurance or logistically complex sports such as skiing:

Pol: Trying to find 2 h a day (to study) was tiring, and I managed to do it, but I felt worn out at the end of the day. And of course, I’d like to think that it didn’t affect my skiing tests, that it didn’t affect my athletic performance, but I don’t know… it was hard. And the exam was hard too, I remember that I managed to pass, but I was angry because I didn’t get a good grade.
Gustau: I have moved up through every category to reach the top team in my club. It’s a very professional level, we train every morning and every afternoon. There are two training sessions a day, and on Saturday we play matches. So that always takes up most of my time.

A lack of foresight and ineffective planning can also result in feelings of stress and frustration:

Martí: I wanted to do more than I could handle, I started to study for my driving test, to do a ski instructor course… and of course, everything came together. Maybe it wasn’t just that the intensity of university and sport had increased, I also wanted to do too many things. I remember that I had so much going on in the first term, I was suffering.

Competencies such as efficient time management and planning of different activities and routines were mentioned as important resources to compensate for the aforementioned barriers:

Carles: You have to be very organized, have everything scheduled, say “now I’m going to do this, then that.” If you have 5 min when you don’t know what to do, this is when you start overthinking and go crazy. This is when you might break down.

The ability to take difficult decisions in order to successfully combine sport and studies also emerged as an important resource:

Joaquim: You have to choose what to do. Whether to go for a stroll with your teammates or use that hour and a half to study. Whether to go out and party or get a good night’s sleep, get up early and be productive. You have to make decisions that are sometimes hard to make, but… I know that the choice is to study or mess things up and have to repeat a subject next year.
Glòria: At the end of the first semester during my second year at university, I wanted to quit the team. […] It was complicated, especially in terms of motivation, feelings… I had realized that they didn’t value me, understand my situation. They only wanted me to do (motorcycle) trials, trials, and trials, and to give up everything else. But they didn’t understand, or didn’t want to understand that I didn’t want to quit studying to devote myself exclusively to the motorcycle world.

The ESAs did not necessarily have the competencies and skills needed to successfully navigate a career in sport and study when they entered university. Rather, they acquired and developed these as they transitioned through the immersion, learning, and adaptation phases:

Gustau: (The second year) I liked it more because I’d already been here for a year, I had made friends here in the faculty, I had met a lot people who helped me, for example, by letting me have their notes, I was already more aware of things, of how everything worked… I had learnt that I had to go and talk to the lecturers, I knew that I had to smarten up and not wait for my tutor to do things for me.
Gerard: I think that in the second year I really learned to work as a team with the people at university. We did a lot of work together and that gave me more confidence in my relationship with others… I maybe didn’t have this level of trust at first.

Some ESAs remarked that belonging to a sports organization with training schedules that were compatible with their academic commitments was an importance resource for finding a balance between athletic, academic, and also personal and social domains (by freeing up time).

Enric: Since I finish classes at 14:30, I have time to eat calmly, I have time to rest, and I have 3 or 4 h that I can use to study or as free time. It’s a different set-up to the one I had with last year’s club, where everything was lumped together, I only had 2 h to organize myself (between classes and training). You could say that things have got better.

In the academic domain, ESAs perceived that their lecturers were sometimes a resource and sometimes a barrier. Whatever the case, the ability to negotiate emerged as an important skill in terms of reconciling academic and athletic obligations:

Cesc: I always explain things, if I have to miss some classes, I’ll mention this in the first few days: “I’m going to miss this, because of that”. And you can see when the lecturer is okay with this, when he or she understands. Obviously, you won’t stop taking exams, you won’t stop handing in assignments… but they adapt. “You can switch classes, do that, let’s do this, come to my office”… You can see that. Then there are others who… (sighs)… who seem to overexert themselves because of you.

One of the main resources mentioned in the academic domain was the dual career assistance program and having a tutor who could mediate with the lecturers.

Glòria: The program helps a lot. Especially as far as missing classes is concerned, there are many lecturers who say “no, your training sessions and travel are included in the 20% of classes you are allowed to miss”. There are a lot of lecturers who don’t understand what competition is all about. In these cases I talk to my tutor and he explains to them that I won’t be able to attend certain classes, and all those things. And on you go.

Another important resource for overcoming academic challenges was support from fellow students:

Cesc: Without my classmates, I would have failed every single subject. Bear in mind that I can’t take notes, I don’t know when assignments are due or when exams have to be taken.

### PASS-Specific Transition Issues

As per Spanish legislation, participants who were officially listed as elite athletes enjoyed important benefits:

Gustau: PASS had a higher percentage of positions reserved for those of us on the BOE (official) lists… if you are in the top six in Europe or the top four in the world, you enter the BOE lists, and you can get into any university degree with a 5.

Some PASS faculties in Spain require students to pass a series of physical tests in addition to meeting the entry grade. Because of their regional or national status as elite athletes, most of the participants in this study were exempt from taking these tests: “I was already an elite athlete because I had represented Spain in some competitions. So I didn’t have to take the physical tests” (Martí). However, participants who did not qualify for this exemption mentioned that the tests were not a problem:

Enric: I only trained for the swimming test. I took advantage of the days I went to the gym with my football team to do extra work and spent the last half hour in the pool. The rest, with my training, was all “standard.”

One of the main barriers associated with combining an elite athlete career and a degree in PASS was the negative impact of practical subjects on training and performance outside the university, as the activities within the degree could cause significant fatigue:

Pol: We used to have sessions to put training theory in practice on Fridays. And I remember that all the sessions involved maximal fitness tests: Cooper, Navette, Léger-Boucher… it was all about endurance. And… (blows loudly) it was pretty brutal. […]. The thing is that it was all on top of the training I had to do anyway, it was like doubling the load. There were weekends when I was completely wiped out, knackered, and I would tell my coach “Look [coach’s name], I don’t know why…”. But actually it was because I had just done a Cooper test to death in class.

Some ESAs also mentioned difficulties with certain practical subjects due to their high level of sport specialization:

Glòria: It may seem strange, but the subjects I found most difficult were the sports subjects […]. For example, I found it really difficult to do team sports, anything that involved catching a ball. Those are the subjects I found most difficult, and those are the ones that you would normally say “well, they are the most fun”, but I liked them the least, and found them to be the hardest.

The participants were also concerned about injury: “I felt really stiff. Of course, some of the sports-based subjects are demanding, almost dangerous, you know? Doing acrobatics… things I don’t usually do to avoid getting injured” (Greta). Nonetheless, all the participants who suffered an injury during the transition period were injured while practicing their own sport, not at the faculty. “With these sports, especially judo, rugby, and handball, you can easily get injured. Obviously, there’s a lot of contact, a lot of sudden movements… But I have been lucky not to get injured” (Joaquim).

It is important to note that injuries were perceived a potential barrier to both athletic and academic development, as students’ grades could be affected if they were unable to perform certain activities:

Raquel: There are always two sides, if you get injured, you hurt the team, you can’t play, you can’t train… But, then, there are your studies. I mean, everything is closely related. If I studied business administration, I could get injured and it would only affect me in sport, but in a degree like this… an injury makes you feel powerless in both areas.

Coaching staff outside the university may facilitate or hinder the successful combination of sport and studies. In an attempt to avoid potentially harmful situations, some of these agents tried to prevent their athletes from doing sport or physical activities at the faculty.

Martí: I remember that he (the coach) wasn’t happy about anything related to contact sports, like judo, rugby, handball… It’s true that you are more likely to get injured in all these sports, he wasn’t happy at all.

Nevertheless, most coaches were sympathetic to the ESAs’ situation and simply requested caution or, on occasions, adapted training loads in accordance with classroom activities:

Raquel: They didn’t restrict us in any way, they know that our degree is what it is. For training they take into account what we are doing at university. For example, on Tuesdays we have practical classes on training theory and we do endurance tests, which are very demanding. And we train on Tuesdays too. The team knows this because we tell them, and they help us a little bit in that they don’t push us so much that day, because they understand that this is what a PASS degree is like.

In some cases, some ESAs chose not to mention to their coaches that they were doing certain activities associated with a risk of injury at university to avoid being restricted:

Pol: If I had an artistic gymnastics exam or something like that… I would not tell him much about that either (laughs). I always think “best not tell him what you’ve done, because you already know what his answer will be.”

The ESAs mobilized different resources to maintain a balance between the physical fatigue caused by their academic and athletic activities and to minimize situations with the greatest potential for injury. For example, they performed university activities with less intensity than required, negotiated a more discreet participation with their lecturers, or counted on the help of fellow students:

Cesc: I haven’t stopped doing things because I have training… Well, at some point, and when I saw that the lecturer understood me better… “well look, today I’m going to stay seated because I have an important competition in 2 days, and it wouldn’t be good for me to be running up and down the track.”

Greta: I played rugby and I liked it a lot. That said I was very careful when tackling and stuff, but when it came to running, feinting, doing the exercises… Here I was not afraid of contact, because my classmates know that I’m an elite athlete and they are very careful.

The ESAs mentioned that in most cases their lecturers were aware of their concerns about injury and about the consequences of becoming injured, and that they also knew that they would sometimes have to miss class or an exam due to their sporting commitments outside university. In general, the lecturers offered the possibility of changing certain academic activities and, within certain limits, they adapted the criteria for evaluating certain subjects.

Enric: I told the sports lecturers with whom I had to do practical classes that I was having an operation, that I would be away for 2 weeks and that I could attend the classes after that but not actually do the exercises or activities until the end of the academic year, because I would have to be in rehabilitation. And they helped me, they said “yes, no problem”. Of course, most of the lecturers on the PASS degree have been elite athletes themselves, they can understand that.

There were also some, albeit fewer, mentions of negative experiences with lecturers:

Greta: I was injured during an emerging sports class […]. The lecturer said “you have to do it, even my grandmother can do it”. And I thought “come on, if the lecturer says this, knowing that as elite athletes we can have these injuries…”. Luckily, a classmate said “don’t worry, I’ll do it for you”. And well, when it came to practicing sport, a simulation of a tournament, I did nothing.

Some subjects in the PASS degree are run intensively over a few weeks and they represented a significant barrier for ESAs who did not have flexible training or competition schedules.

Carles: I wasn’t able to participate in the intensive outdoor activity week. Not because I was injured, but because I couldn’t afford to miss a week (of training).

The close links between the ESAs’ athletic activities outside the university and the content of the degree was highlighted as a significant resource in terms of academic progress. However, it should be noted that the PASS degree has a multidisciplinary curriculum combining knowledge in high-performance sport, health, physical education, sports management, and leisure. Consequently, perceptions of the ease or difficulty of a particular subject varied according to individual preferences:

Joaquim: It might sound like a cliché, but it’s easier to study what you like than what you don’t. For example, last year I loved sports psychology and motor skills. They’re subjects I really like, that have to do with education, and I found it easy to study them, to learn them. On the other hand, in secondary school, subjects like Catalan, Spanish, English… all that was much more difficult for me (Table 2).

**TABLE 2:** Main resources and barriers perceived.

	**Levels of development**	**Resources**	**Barriers**
General university transition issues	Athletic	Sports organizations with favorable training schedules	Competing and training at more than one level at the same time (junior and senior) Higher athletic demands
	Psychological	Time management and planning Ability to take difficult decisions Ability to negotiate	Feelings of stress, frustration Lack of time to study Lack of foresight, ineffective planning
	Psychosocial	Support from fellow students	
	Academic-vocational	Supportive lecturers Dual career assistance program	Greater academic demands Work commitments Unsupportive lecturers
	Financial	Acknowledgment of impossibility of making a living from sport	
PASS-specific transition issues	Athletic		Sport: overtraining, risk of injury
	Psychological	Ability to negotiate Taking it easy with the practical subjects	
	Psychosocial	Supportive coaching staff Support from fellow students	Unsupportive coaching staff
	Academic-vocational	Close link between sport and content of the degree Favorable legislation Majority presence of former (elite) athletes or other kinds of sports professionals among teaching staff	Practical subjects: overtraining, risk of injury Intensive nature of some subjects (e.g., week of outdoor activities)

### Transition Pathways

An empirical taxonomy of the transition pathways (i.e., a taxonomy grounded in empirical data) was identified based on the experiences reported by the ESAs and the priority they assigned to their athletic and academic roles. Three types of pathways were observed. In the first group, the ESAs followed a convergent path where they showed greater commitment to sport than education:

Gustau: Some people put their degree before sport, they miss many of the morning training sessions. But I’ve always put training before studying, but at the same time I try to do as much as possible for the degree.

Elite student-athletes on this convergent path perceived greater difficulties associated with the transition to university, although most of them successfully overcame these difficulties, mainly because they were studying a degree that was closely linked to their athletic activities:

Greta: I want to do things very well, but I can’t spend as much time on my studies as I would really like to. It is like… I know I could do better, and I’m not doing better because I have a very time-consuming sport, and this is my priority.

Martí: The pressure starts to increase when you start university. But the truth is that, despite being the first year… as there are many practical subjects, there are many sports… maybe that is why it didn’t feel so hard, because I like it.

Nevertheless, despite attaching greater importance to the athletic domain, ESAs who chose this path showed conviction in terms of continuing their studies, as they were aware that having a university degree would be an important asset for them when it came to retiring from sport and pursuing other social and occupational activities.

Gustau: If you are involved in a minority sport, or are not competing at a very, very high level… having an ADO scholarship (scholarship from the Spanish Olympic Sports Association) and similar stuff that allow you to keep going, that allow you to live… well, you have to smarten up and continue with the degree. If I don’t get a degree, what will become of me?

The second transition path followed was the parallel or balanced path, characterized by perceived equal commitment to the academic and athletic domains. This identity balance was indirectly evident in a number of comments, such as the one below, which also shows how strong commitment to both domains gives rise to feelings of stress:

Gerard: I felt very bad inside… I was always thinking “now I have to train, but I should also study, I can’t just be training, I have to study and be able to go to university”. This feeling of… if I was not able to do both things at the same time, that meant I would have to give up one or the other. This feeling of “I don’t want to give either up, because I like them both”… I wanted to continue as far as I could.

The third path was a convergent path in which ESAs attached greater priority to their studies than to sport:

Joaquim: I have always thought that education was more important than sport. And if I am here, if I have come all the way from my home town to study this degree, then sport becomes secondary, doesn’t it? If they told me “either you stop taking volleyball at university or we will not call you anymore, you will stop playing”… I would say that I’m not going to play with the hockey team anymore. I would either quit, or look for something else, I’m sure that there are other teams that would accept me and not cause any problems. But I would never give up a university subject.

In the cases mentioned above, the ESAs showed stable identities and commitments to the paths they had chosen. We also, however, identified what we term “fluid transition paths” in which ESAs attached varying importance to sport and studies depending on the situation. The following quotes provide examples of these trajectories. In the first case, the change was related to an opportunity to advance the athlete’s career in a short period of time:

Martí: This year I have a more sports-oriented goal, I am looking for a big improvement. Because this year the World Roller Games are being held here in Barcelona. […] It is a very important competition within the world of skating, within my sport. It’s a kind of skating “Olympics”, with 11 disciplines. And well, I decided that, since I’d performed well last year, had good results… I want to go one step further. […] This year I am focusing more on the sports part.

We also observed that qualitative improvements in athletic performance and the consequences of these improvements, such as moving to a new club with a greater chance of success, can also change priorities:

Raquel: It’s true that maybe I had stopped training so I could study, but that is on a general level, ok? In the final year of secondary school and during the university entrance exams. Because well, obviously at that time my studies were more important. In my case, studies have always come before sport. But now that I’m competing at this level, it could be said that the scales have been tipped to an equal position between education and sport.

The overlapping of transition to higher education and transition from junior to senior competition can also influence dual career paths, particularly for ESAs who perceive that the demands of the athletic transition cannot be overcome or are not worth overcoming.

**FIGURE 1:**
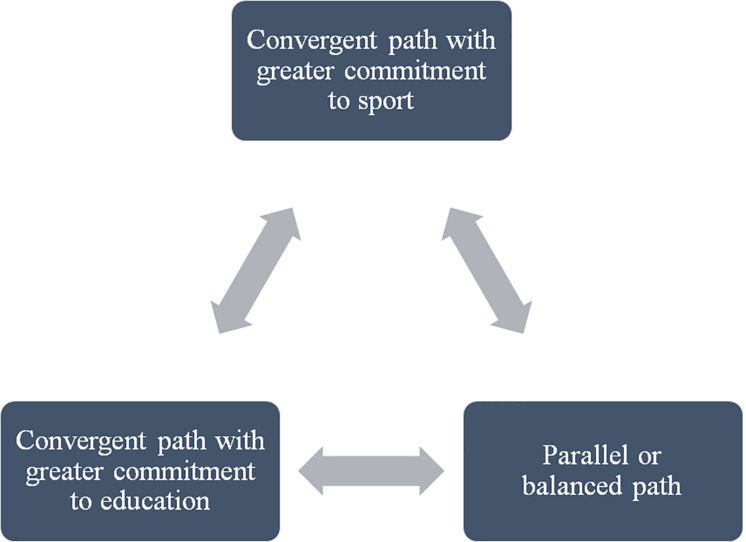
Types and fluidity of transition pathways.

Pol: The first and second years (at university) were the most conflictive in this regard, because I really, really, really focused on competition then. Now in the third year I have tried to prioritize my studies a little more, although I am still on the senior Catalan team. But I do try to prioritize my studies more (Figure 1.

## Discussion

To our knowledge, this is the first study to analyze dual careers within the framework of a university degree in PASS. The first aim of the study was to investigate the main perceptions associated with demands, barriers, and resources among ESAs during various phases of their transition from secondary school to a university degree in PASS. The second aim was to identify the transition pathways followed by the ESAs depending on the subjective importance they attached to sport and education. The main findings of our analysis showed that the close link between sport and the content of the degree was perceived as the participants’ most relevant resource for overcoming the transition and continuing with their dual career. By contrast, the increased physical demands resulting from the practical side of the degree, together with other factors, such as the transition from junior to senior competition (e.g., higher training loads), represented the most significant barrier, as the risk of injury and overtraining had potentially negative effects for both academic and athletic performance. Lastly, we identified a taxonomy of the transition pathways pursued and observed variations in the importance the ESAs gave to the athletic and academic domains as they moved through the transition process. We termed this phenomenon “fluid transition pathways.”

The resources mentioned by the ESAs in the athletic domain have already been identified in previous research and included belonging to a sports organization with compatible training schedules and studying, training, and living in close proximity (Guidotti et al., 2015; Guirola et al., 2016; Pérez-Rivasés et al., 2017). Although transition to higher education usually coincides with the transition from junior to senior competition, which in itself is a challenge (Torregrossa et al., 2016), we found that the ESAs interviewed had differing perceptions of their transition to university. The greatest barrier to pursuing an athletic career and completing a degree in PASS was having to do practical subjects as part of the degree as, supporting reports by Honta (2007), these were associated with a greater risk of suboptimal athletic performance and injury.

The participants showed a range of skills within the psychological domain that helped them cope with the demands of transitioning from school to university, supporting findings of several European studies that have highlighted the importance of applying and improving these (and other) skills to achieve optimal dual career development among elite athletes in higher education. Key skills and competencies linked to successful dual career management (De Brandt et al., 2018), include self-discipline, effective time management, the ability to set priorities (Petrie and Stoever, 1997; Geraniosova and Ronkainen, 2015; Miró et al., 2018; Brustio et al., 2019), and career planning and exploration (Torregrossa et al., 2015; De Brandt et al., 2018; Miró et al., 2018; Gavala-González et al., 2019). In our series, most of the ESAs were already thinking about employment opportunities after university and after retirement from sport, and this was an important motivator for them. We also observed social intelligence competencies (De Brandt et al., 2018), such as the ability to communicate and negotiate with relevant agents in the academic and athletic environments (Aquilina, 2013) and the ability to seek and accept help from friends and/or classmates (Miró et al., 2018). Finally, we observed emotional competencies, such as the ability to manage stress due to the double demands of academic and athletic commitments (De Brandt et al., 2018). Our research shows how the ESAs gradually acquired some of these competencies as they progressed through the different phases of their transition, possibly explaining why the preparation and immersion phases were perceived as more challenging than the learning and adaptation phases (Giacobbi et al., 2004; Pérez-Rivasés et al., 2017). Brown et al. (2015) call this process “transition toward personal responsibility,” which occurs as an ESA accumulates experiences at university and learns the necessary codes, mechanisms, and competencies to progress in this setting. The main barriers that were identified were a lack of time, overlapping of academic and athletic schedules, high expectations in both these domains, and resulting stress (Giacobbi et al., 2004; Brown et al., 2015). Acquisition of stress and expectation management skills (De Brandt et al., 2018) can be essential, as even with good planning and organization, it is not easy to perform well as both an athlete and student (Gavala-González et al., 2019). Faced with the greater time investment required in both domains, most ESAs chose to prioritize the athletic domain, leaving them less time to study (Gavala-González et al., 2019). Contrasting with previous findings (McKenna and Dunstan-Lewis, 2004; Gavala-González et al., 2019), none of the ESAs in our study considered quitting their career as an athlete because of the demands they faced during transition to university.

In the psychosocial domain, the ESAs perceived their classmates as essential agents for facilitating the transition to university, supporting previous findings (Miró et al., 2018; Gavala-González et al., 2019). Understanding and support from the ESAs’ clubs and coaching staff, and interest in what they were doing, also was an important resource for balancing athletic and academic commitments (Giacobbi et al., 2004; Brown et al., 2015; Guirola et al., 2016). There was also a minority group of ESAs who perceived that sports agents sometimes acted as barriers, showing that there is still a tendency in certain fields and/or in certain organizations to objectify their athletes, to see them as mere results-oriented commodities rather than complex individuals who feel, think, and develop in other life domains besides their sport (Barker-Ruchti et al., 2016). Finally, ESAs who moved far from their homes to study or train experienced more barriers in the psychosocial field, in particular feelings of loneliness, which were experienced with greater intensity in the immersion and learning phases, supporting previous findings (Tracey and Corlett, 1995; Gavala-González et al., 2019).

In the academic domain, the Spanish legislative framework for facilitating access to a university education for elite athletes was perceived as an important resource, again supporting previous findings (Aquilina and Henry, 2010). Other researchers in Europe have reported that participation in a dual career assistance program is a key resource throughout the transitional period, as it helps participants balance their academic and athletic commitments (Stambulova and Wylleman, 2014; Brown et al., 2015; Geraniosova and Ronkainen, 2015; Mateu et al., 2018b). Tutors as both counselors and mediators are an essential part of these programs (McKenna and Dunstan-Lewis, 2004; Mateu et al., 2018b). Nevertheless, it should be noted that at our university, the faculty’s teaching staff is not obliged to participate in the dual career assistance program, as lecturers’ autonomy prevails over initiatives of this nature in the Spanish university system. Thus, even though most of the lecturers offered flexibility and were sympathetic to the ESAs’ situation (Brown et al., 2015; Guirola et al., 2016), conflicts and disagreements may occasionally arise between teaching staff and ESAs (Mateu et al., 2018a). While tutors can mediate in such situations, we also consider it important for ESAs to develop communication and negotiating skills to be able to manage potential conflicts themselves. Finally, while the similarity between the ESAs’ athletic activities and the content (and applicability) of the PASS degree was highlighted as a resource (Lupo et al., 2017a), the risk of training overload and injuries was shown as a potential major barrier to normal dual career development, supporting reports by Honta (2007).

In the financial domain, as most ESAs could not afford to make a living from their athletic activity, in either the short or long term (Guirola et al., 2016), they accepted that their education was important for their future. This would explain why even those with a stronger athletic identity showed commitment to their studies (Geraniosova and Ronkainen, 2015; Gavala-González et al., 2019). We therefore understand that the ESAs’ limited capacity to accumulate economic capital from their athletic careers motivated them to pursue a dual career.

All the ESAs were very interested in succeeding both as an athlete and a student, which coincides with previous research studies such as that conducted by Lupo et al. (2017b) and Brustio et al. (2019). However, they chose different paths to achieve their goals (McKenna and Dunstan-Lewis, 2004), which is consistent with the work of Miró et al. (2018) and Pallarés et al. (2011) and, in our opinion, would suggest that the ESAs had multidimensional identities characterized by different degrees of perceived priority attached to the academic and athletic domains. Furthermore, our results suggest that differing subjective importance attached to athletic and academic domains was not a determinant of perceived transition difficulties, possibly because of the already mentioned similarity between the content of the PASS degree and the existing knowledge and skills of the athletes (Honta, 2007). We also observed that different transition paths and hence different levels of commitment to academic and athletic domains can vary over time. We have called this phenomenon “fluid transition paths.” Our position thus coincides with that of Hodkinson and Sparkes (1997) in that we do not understand ESA careers as trajectories, in the more deterministic sense of the word, with clear start and end points, but rather as paths with different forks and events that affect decisions and life priorities over time.

We have addressed several aspects associated with transitioning from secondary school to university among a population of Spanish ESAs enrolled in a PASS degree. By providing a better understanding of this complex process, we hope not only to promote reflection in the academic environment but also to encourage the development of measures (establishment and/or improvement of dual career assistance programs, collaboration between universities and clubs, etc.) that could provide ESAs with a better chance of successfully developing and completing a dual career by enhancing the resources available to them and limiting, in so far as is possible, the barriers detected. This goes beyond just providing better academic schedules, which seems to be a good solution for the combination of sport and studies in the general group of Southern European elite student-athletes (Brustio et al., 2019). In the case, for example, of the obligation to complete certain practical subjects as part of the PASS degree, alternative forms of evaluation could perhaps be considered to help overcome this perceived barrier. In this regard, synergies between dual career assistance programs and other university initiatives for students with special education needs could be enhanced. Likewise, we recommended that young elite athletes still in secondary education, together with career guidance counselors, carefully weigh up the benefits and risks associated with pursuing a degree in PASS or similar. And finally, the development of key competences mentioned above (e.g., time management, expectation management, career planning, and stress management) is also an area on which sport psychologists and/or dual career support providers can focus in order to provide elite student-athletes with significant resources to deal with their transition.

The main limitation of our study, like most studies of the transitional experiences of elite athletes, is its retrospective design (Alfermann and Stambulova, 2007). This design means that there was only a single answer available for analysis and also entails a risk of recall bias. Perhaps a longitudinal study, such as the ones carried out by Torregrossa et al. (2015) or Pérez-Rivasés et al. (2017), would have enabled a more detailed analysis of the phenomena studied and their respective complexities. Researchers should continue to investigate transitions to specific university degrees among elite athletes, but should ideally include several data collection points that reflect different moments in time. Different types of universities (e.g., public and private) might also be considered, as in Spain there are important differences between universities in terms of dual career assistance services as academic flexibility or economic support (López de Subijana et al., 2014). It would also be interesting to investigate the experiences of people who are forced to quit their academic or athletic careers during this transition period because they were unable to meet their dual obligations.

## Conclusion

We have described, for the first time, the experiences of ESAs during their transition from secondary education to a university degree in PASS. Through a thematic analysis of transcripts from semi-structured interviews, we identified a number of perceived demands, barriers, and resources. Having to perform at times risky or strenuous tasks as part of the practical subjects on the curriculum was perceived as an important barrier that could potentially impact both athletic and academic performance. It was not, however, perceived as being sufficiently serious to lead any of the ESAs to contemplate quitting either activity. The empirical taxonomy of the transition pathways pursued by ESAs studying a university degree in PASS, together with the fluidity observed between these pathways, is an important contribution of this study to the literature and our understanding of dual careers and educational transitions in this setting. Based on the above findings, we propose that educational institutions and/or sports organizations design or improve existing initiatives to facilitate the successful combination of an elite career in sport and successful completion of a university degree that is highly sought after by elite athletes in Spain.

## Data Availability Statement

The datasets generated for this study will not be made publicly available. Complete interview transcripts could make participants identifiable. Requests to access the datasets should be directed to the corresponding author.

## Ethics Statement

This study was performed in compliance with the Declaration of Helsinki and was reviewed and approved by the Ethics Committee for Clinical Research of the Catalan Sports Council. Written informed consent was obtained from all participants before the interviews and each participant was treated in accordance with the ethical principles of respect, confidentiality, and anonymity. The provisions of the Spanish Organic Law 15/1999, of December 13, on Personal Data Protection were met during and after the study.

## Author Contributions

PM, AV, EI, and MT: study design. PM: data collection. PM, AV, EI, MT, and RM: data analysis and interpretation. MT and NS: critical friends. PM, AV, EI, MT, RM, and NS: manuscript preparation. AV: conceptualization and funding procurement. All authors contributed to the article and approved the submitted version.

## Conflict of Interest

The authors declare that the research was conducted in the absence of any commercial or financial relationships that could be construed as a potential conflict of interest.

## Footnotes

1 In this study, the term “elite athlete” is used as a generic term to refer to an athlete who trains and competes at a high level. We included all types of elite athletes covered by the PASS faculty’s dual career assistance program: “high-level athletes” and “high-performance athletes” as per the Spanish Royal Decree 971/2007, 2007 and “Catalan high-level athletes” as per the Catalan Decree 337/2002. Professional athletes, i.e., athletes with an employment relationship with a sports clubs and/or another organization, were included in this definition as well.
